# Supplementary material for: Polarizability-Dependent Sorting of Microparticles Using Continuous-Flow Dielectrophoretic Chromatography with a Frequency Modulation Method
Source: Micromachines (Basel). 2019 Dec 28;11(1):38. doi: 10.3390/mi11010038 (PMC7019690; doi:10.3390/mi11010038)
Supplement: Supplementary file 1 [file micromachines-11-00038-s001.zip › micromachines-650056-supplementary materials.pdf]

micromachines

Article

Supporting Information: Polarizability-Dependent Sorting of Microparticles Using Continuous-Flow Dielectrophoretic Chromatography with a Frequency Modulation Method

Section S1. Method

Figure S1 shows the Re(*CM*) and mobility with respect to the frequency. Since the surface conductance (here: $K_s = 1$ nS) is not easily measurable, these curves were assumptions and were used as a starting point for process parameters. For constant surface conductance and medium conductivity, the Re(*CM*) only depends on particle size. The chosen frequency band should include the cross-over frequency of all particles to generate nDEP and pDEP.

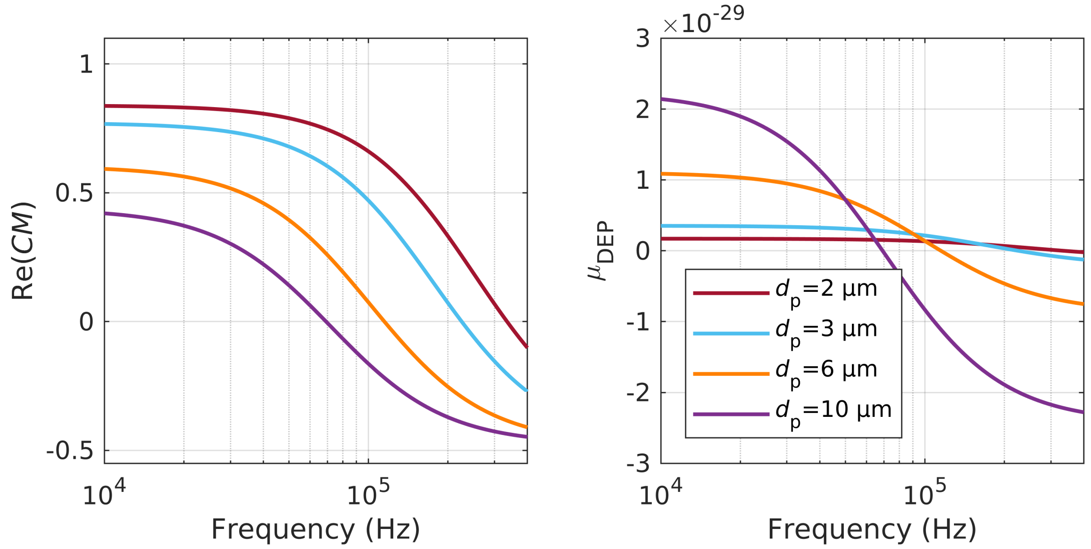

Figure S1. Real part of the Clausius–Mossotti factor and dielectrophoretic mobility over frequency of the electric field of polystyrene particles ($d_p = 2$ μm, 3 μm, and 6 μm). Particles suspended in DI water as the surrounding medium, with $\sigma_m = 1.2$ μS cm^{-1} and $\mathcal{E}_m = 78.5$).

Section S2. Device Fabrication

2.1. PDMS Device

The fabrication of PDMS microfluidic devices (soft lithography) is a well known process, which was described in the literature before [1,2]. A SU8-3050 master mold was produced using a laser printed mask (25,000 dpi KOENEN GmbH, Germany, Figure S2 b) and standard photolithography techniques. Afterwards, the PDMS channel could be made by pouring well mixed and degassed PDMS (10:1, base: curing agent, Sylgard 184 Dow corning) onto the master mold. After curing the PDMS at 80 °C for 1 h in a convection oven, the polymer can be cut into pieces as desired using a scalpel. The inlet and outlet, both having an inner diameter of 1.5 mm, were punched using biopsy punches (Rapid-Core, World Precision Instruments, Inc.).

2.2. Electrodes

The electrodes were fabricated using physical vapor deposition of first chrome as the adhesion layer and second gold as the final layer. As the substrate, 100 mm borosilicate glass wafers were used. Afterwards, using a chrome mask (100,000 dpi bvmmask shop, Germany), positive photoresist, followed by a wet etching process, the final electrodes were obtained. These electrodes had a width of 100 µm and an identical gap width. To connect the amplifier to the flow cell, contact pins were soldered onto the electrode. The full electrode chip had a length of 58 mm and a width of 26 mm (Figure 2 a)

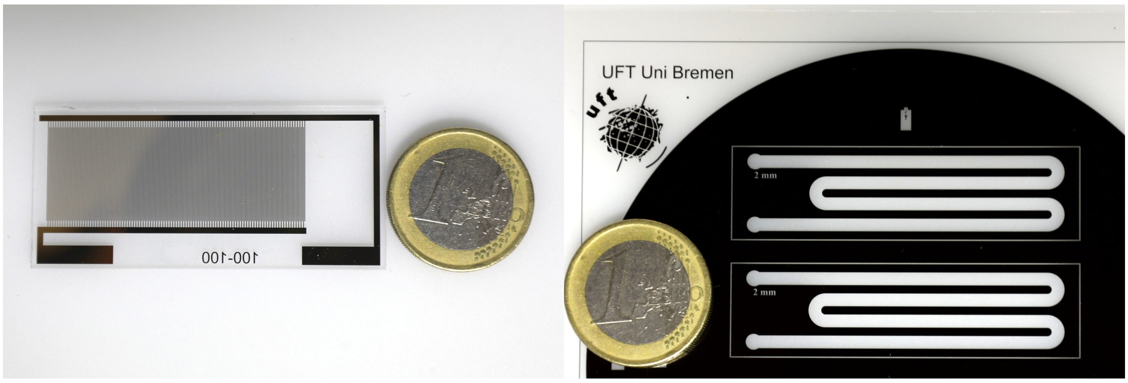

a) b)

Figure S2. (**a**) Photograph of the gold electrodes used with a 100 µm electrode width and gap width. (**b**) Photomask for producing the SU8 master mold.

Section S3. Post-Processing

A segmentation algorithm was used to extract the fluorescence intensity of each color of fluorescence into different images [3]. Afterwards the intensity of each image was calculated, and a moving average was applied to reduce the level of noise of the measured intensities. Finally, the background signal was subtracted for each color to eliminate scattered light and permanently adhered particles from the data. Since all measurements produced slightly different maximum values and each particle class had a different fluorescence intensity per particle, the data were normalized by the maximum intensity value of each experiment.

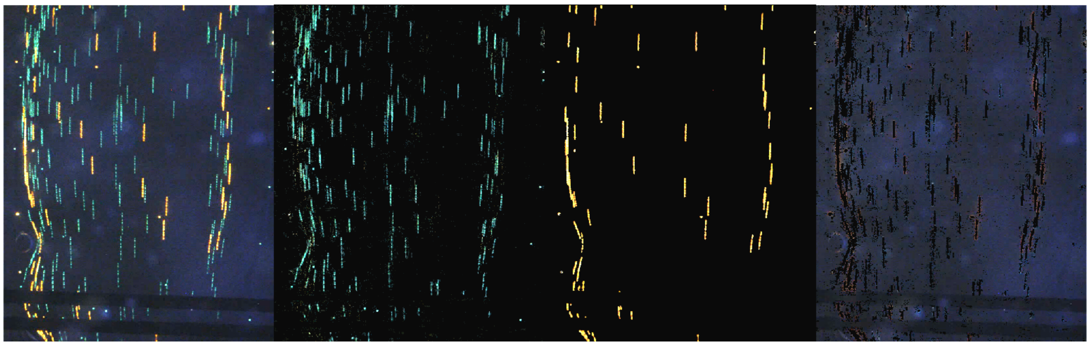

Figure S3. Result of the segmentation process. Original Image, Particle Type 1, Particle Type 2, and background (from left to right).

Section S4. Further Experimental Results

4.1. Full Set of Intensity Profiles

4.1.1. Separation of 6 and 3 μm Particles

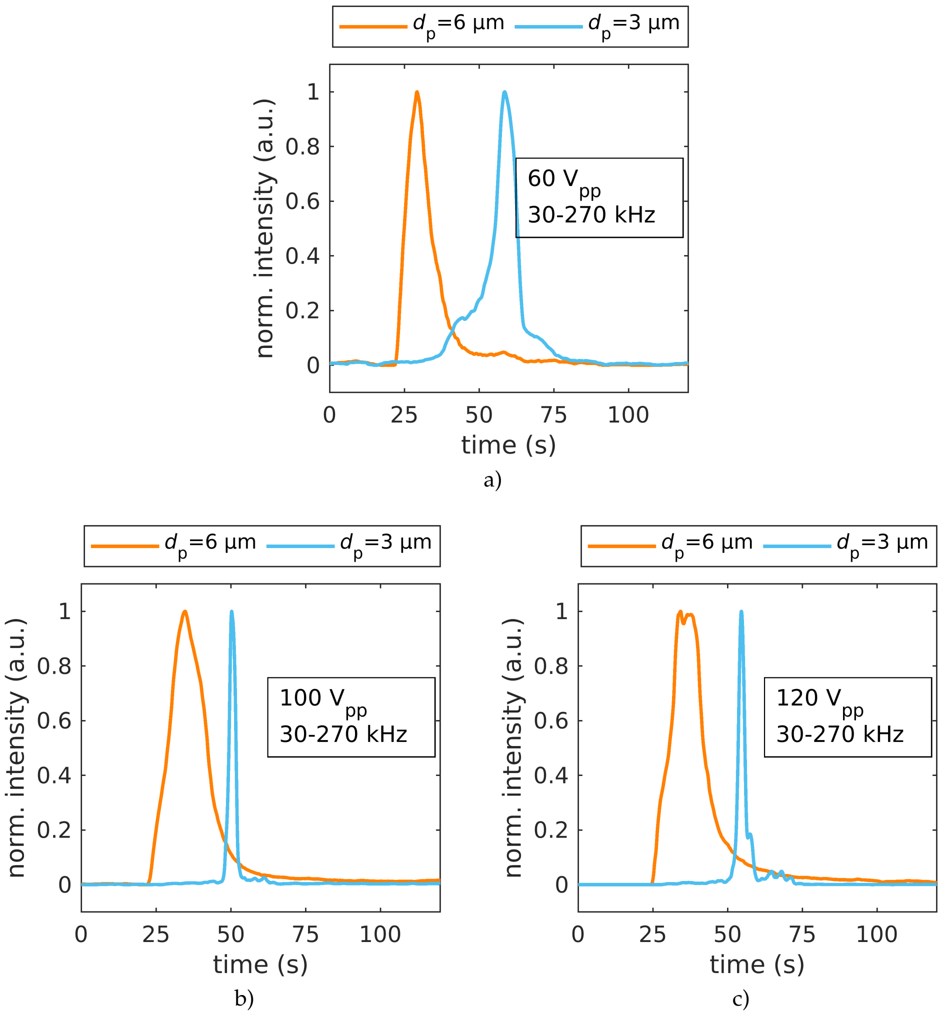

Figure S4. Fluorescence intensity over time of 3 μm and 6 μm fluorescent polystyrene particles at (**a**) 60 V_{PP}, (**b**) 100 V_{PP}, and (**c**) 120V_{PP}; all experiments at 30 kHz–270 kHz with a modulation frequency of 300 MHz.

The separation of 3 μm and 6 μm fluorescent polystyrene particles at all voltages not shown in the main manuscript is show in Figure S4. For all applied voltages, almost no retardation of the 6 μm particles could be observed, whereas for 3 μm, the residence time diverged significantly from experiments without applied voltage. According to our calculations, this separation was due to the retardation of the 3 μm particles, whose behavior was dominated by pDEP.

4.1.2. Separation of 6 and 10 µm Particles

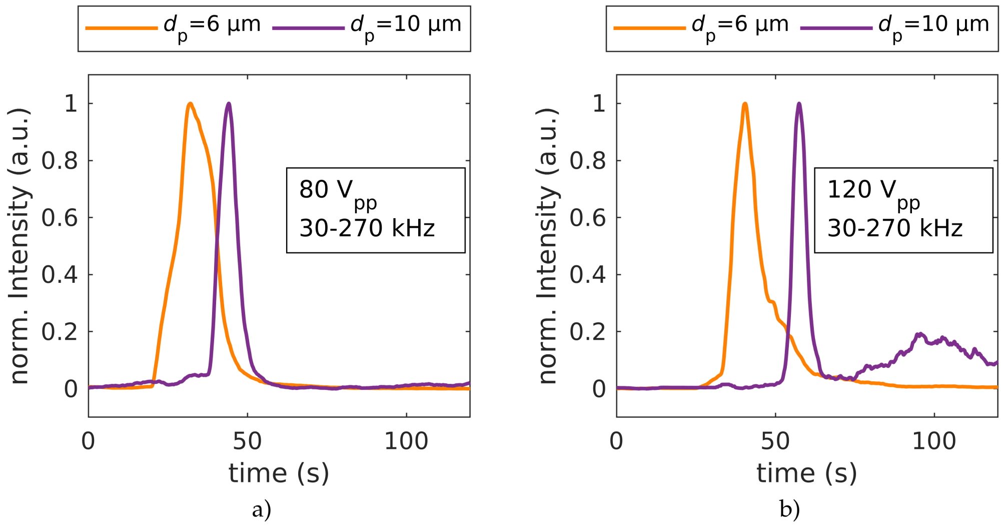

Figure S5. Fluorescence intensity over time of 6 µm and 10 µm fluorescent polystyrene particles at (**a**) 80 V_{PP} and (**b**) 120 V_{PP}; all experiments at 30 kHz–270 kHz with a modulation frequency of 300 MHz.

To investigate the retention time of particles, which showed, according to the literature, substantially more nDEP than pDEP, the separation of 6 µm and 10 µm was chosen. In addition to the data presented in the main document, the separation at 80 V_{PP} and 120 V_{PP} is presented in Figure S5.

4.1.3. Separation of 2 and 3 µm Particles

The increase in resolution while increasing the applied voltage of the separation of 2 µm and 3 µm fluorescent polystyrene particles is illustrated in Figure S8. With increasing voltage, the width decreased, and the peak maxima diverged progressively.

The variation of the set of frequencies (30 kHz–270 kHz vs. 80 kHz–320 kHz) is illustrated in Figure S7 for 160 V_{PP}. The decrease in smaller retention time using 80 kHz–320 kHz is shown in Figure S6. The particles eluted significantly later, when 30 kHz–270 kHz were applied. The higher retention time, when using 30 kHz to 270 kHz, was due to less negative dielectrophoretic movement. Therefore, the particles were more likely to stay in areas near the electrode in which only low fluid velocity was present.

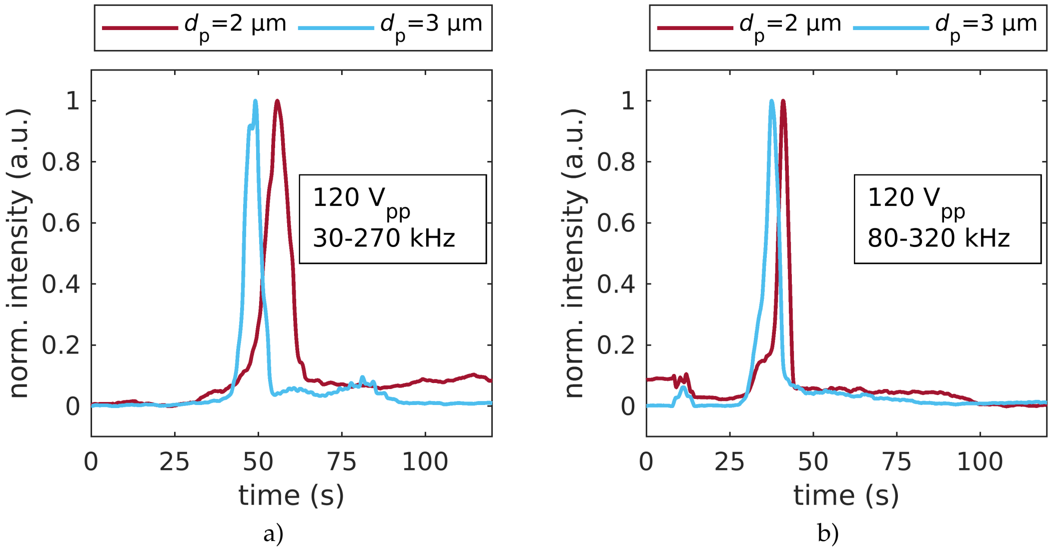

Figure S6. Fluorescence intensity over time of 2 µm and 3 µm fluorescent polystyrene particles: (**a**) 120 V_{pp} at 30 kHz–270 kHz and (**b**) 120 V_{pp} at 80 kHz–320 kHz with a modulation frequency of 300 MHz.

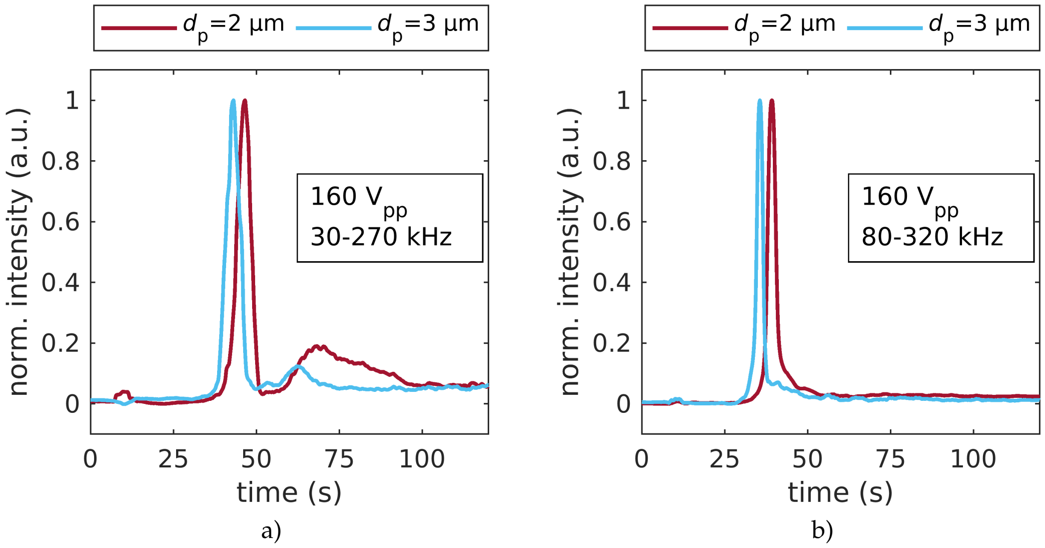

Figure S7. Fluorescence intensity over time of 2 µm and 3 µm fluorescent polystyrene particles: (**a**) 160 V_{pp} at 30 kHz–270 kHz and (**b**) 160 V_{pp} at 80 kHz–320 kHz with a modulation frequency of 300 MHz.

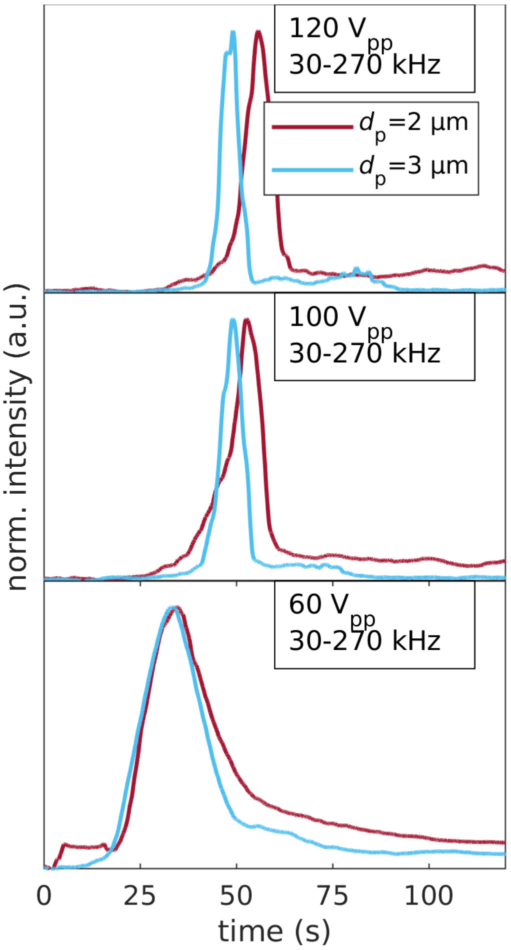

Figure S8. Fluorescence intensity over time of 2 µm and 3 µm fluorescent polystyrene particles using different voltages. With increasing voltage, the separation resolution R_s increased from R_s(60 V_{pp}) = 0.051 ± 0.14 to R_s(100 V_{pp}) = 0.24 ± 0.07 and finally to R_s(120 V_{pp}) = 0.54 ± 0.1 (N = 4 experiments).

4.2. Videos of Separation and PIV Data

4.2.1. PIV of 10 µm Particles

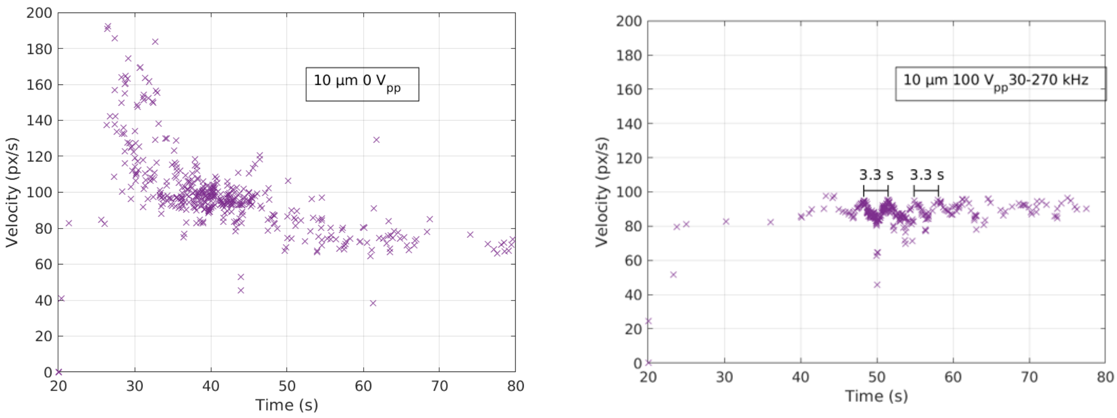

a) b)

Figure S9. PIV data of 10 µm PS particles at (**a**) 0 V_{PP} and (**b**) 100 V_{PP} at 30 to 270 kHz with a modulation frequency of 300 MHz. The measurement was performed at the outlet of the DPC channel, where no electrodes are present.

We conducted experiments with 10 µm at 0 V_{PP} and 100 V_{PP} to perform PIV measurements utilizing ImageJ and the TrackMate algorithm (see Figure S9). Without applying an electric field, at first, fast particles arrived, whereas with increasing time, slower particles eluted from the channel. This was due to the laminar flow profile of the microchannel. When a voltage was applied, the velocity became more homogeneous and slower. The particles eluted later from the channel, and their velocity fluctuated with a 3.3 s period, which again was the cycle length of the frequency modulation. The fluctuation had a peak-to-peak value of about 15 px/s (80 to 95 px/s). The measurements were conducted at the outlet of the channel close to the last electrode the particles passed in the channel. No such periodic velocity change was observable for the 6 µm particles (which were still big enough to perform PIV measurements). We also added a Video S1 "10um_100Vpp_PIVdata.mp4" to the Supplementary Information from which the PIV data were evaluated. However, the periodic velocity changes were too small to observe them with the naked eye.

4.2.2. Videos for 3 µm and 6 µm Particles

For smaller particles, no PIV measurements were possible, due to their low fluorescence intensity. However, videos (0 V_{PP}: Video S2 "DPC_0Vpp_3vs6umPS.mp4" and 80 V_{PP}: Video S3 "DPC_80Vpp_3vs6umPS.mp4") are available as Supporting Documents of the separation of 3 and 6 µm particles with subtitles in it to guide the eye. The videos are cropped and adjusted in contrast, color, and brightness for better visibility. For future work, a more powerful light source/laser could be used to generate a more detailed set of PIV data. These videos clearly show how the 3 µm particles slowed down in intervals that matched the applied frequency modulation. The videos were taken at the outlet of the channel close to the last electrode the particles passed in the channel.

1. Tu, 79 J.; Qiao, Y.; Feng, H.; Li, J.; Fu, J.; Liang, F.; Lu, Z. PDMS-based microfluidic devices using commoditized PCBs as masters with no specialized equipment required. RSC Adv. **2017**, 7, 31603–31609.doi:10.1039/c7ra03899b.
2. Faustino, V.; Catarino, S.O.; Lima, R.; Minas, G. Biomedical microfluidic devices by using low-cost abrication techniques: A review. J. Biomech. **2016**, 49, 2280–2292. doi:10.1016/j.jbiomech.2015.11.031.
3. MathWorks. Color Based Segmentation Using the L * a * b * Color Space, 2019.

© 2019 by the authors. Submitted for possible open access publication under the terms and conditions of the Creative Commons Attribution (CC BY) license (http://creativecommons.org/licenses/by/4.0/).
